# Comparing the Efficacy of Solely Scapular Stability Training versus Combined Thoracic Flexibility Training in Elite Table Tennis Players with Scapular Dyskinesis

**DOI:** 10.5114/jhk/196512

**Published:** 2025-05-29

**Authors:** Yijun Lin, Ruike Zhang, Yifan Zhang, Yang Xu, Qi Gao, Qinglu Luo

**Affiliations:** 1 Rehabilitation Medicine Center and Institute of Rehabilitation Medicine, West China Hospital, Sichuan University, Chengdu, China.; 2 Key Laboratory of Rehabilitation Medicine in Sichuan Province, West China Hospital, Sichuan University, Chengdu, China.; 3 Department of Rehabilitation Medicine, Key Laboratory of Biological Targeting Diagnosis, Therapy and Rehabilitation of Guangdong Higher Education Institutes, The Fifth Affiliated Hospital, Guangzhou Medical University, Guangzhou, China.; 4 School of Sports Medicine and Rehabilitation, Beijing Sport University, Beijing, China.; 5 Department of Rehabilitation Medicine, The Tenth Affiliated Hospital of Southern Medical University (Dongguan people's hospital), Dongguan, China.; 6 Dongguan Experimental Centre for Sports Rehabilitation Research, Dongguan, China.; 7 Dongguan Key Specialty of Traditional Chinese Medicine (Rehabilitation Department), Dongguan, China.

**Keywords:** racquet sports, scapular dysfunction, thoracic spine flexibility

## Abstract

This study aimed to compare the effects of scapular stability training (SST) alone and combined scapular stability and thoracic flexibility training (TFT) in elite table tennis players with scapular dyskinesis (SD). Thirty players were categorized into three groups based on the presence of scapular dysfunction: the control (n = 10), shoulder (n = 10, with dysfunction), and chest groups (n = 10, with dysfunction). The shoulder group underwent scapular stability training alone, while the chest group underwent combined scapular stability and thoracic flexibility training. Various variables, including average track error (ATE), angular velocity, lateral scapular slide test (LSST) positions, upper quarter Y-balance test (YBT) distance, and range of motion (ROM) of thoracic rotation, were measured before and after the interventions. For the shoulder group, the playing-side ATE decreased significantly after the experiment (p = 0.005), along with increased angular velocity. LSST positions one, two, and three were smaller after the experiment (p values of 0.008, 0.008, and 0.009, respectively), indicating improved proprioception. The chest group exhibited significant differences in playing-side ATE, angular velocity, LSST positions two and three, YBT distance, and ROM thoracic rotation after training (all p < 0.05). The playing-side YBT distance (p = 0.002), ROM of thoracic rotation to the right (p = 0.001), and angular velocity (p = 0.003) were all higher in the chest group than in the shoulder group. Both interventions positively impacted scapular stability, but combined training, especially in the chest group, showed additional benefits in terms of angular velocity, YBT distance and thoracic rotation. These findings suggest that combined scapular stability and thoracic flexibility training may offer superior outcomes in elite table tennis players with SD.

## Introduction

The large range and flexibility of the shoulder joint are essential for power and accuracy in table tennis, but they can also cause adaptive changes and increase the risk of injury, especially in the elbows and shoulders of athletes (Kamonseki et al., 2018; Shang et al., 2022; Zhang et al., 2020). One-fifth of players have shoulder problems as a result of the strain that intense training puts on their shoulders, leading to pain and injuries. Moreover, a prevalent issue is scapular dyskinesis (SD), which elevates the risk of shoulder pain by 43% (Burn et al., 2016). In general, there is a high incidence of SD and shoulder pain in table tennis players (Teo et al., 2021).

Shoulder injuries are often related to scapular movement dysfunction (Sungkue et al., 2024), affecting the scapular position and motion, potentially weakening the rotator cuff muscles, causing impingement, and reducing the size of the subacromial space (Mihata et al., 2010). The quick and repetitive motion of table tennis can injure rotator cuff muscles, which are essential for maintaining scapular stability (Li et al., 2021; Zhang et al., 2020). However, the impact of SD treatments on scapular posture and motion needs clarification (Kibler et al., 2013; Nodehi Moghadam et al., 2020).

Swinging, sprinting, and turning are key table tennis movements requiring coordinated effort (Gu et al., 2019); thus, training should address strength, power, and coordination (Zagatto et al., 2018). Agility and coordination are closely linked in ball sports, with muscle stability being essential for quick, efficient movement (Sekulic et al., 2013). Moreover, scapular stability exercises can lessen pain and improve SD in patients with subacromial impingement syndrome (SIS), as shown in a systematic review with 228 participants (Ravichandran et al., 2020). Strength training, especially for rotator cuff muscles, and closed-chain stability training are beneficial for shoulder stability (Zhang et al., 2020). Scapular-focused exercises are effective at treating shoulder pain and improving function (Kamonseki et al., 2023), and stabilizing muscles is vital for shoulder movement coordination (Tooth et al., 2020). Therefore, shoulder stability training is a primary method for alleviating joint pain and SD in players.

Nevertheless, attaining the best possible therapeutic results for SD is still challenging since therapists frequently ignore the entire biomechanics of the shoulder joint during treatment. Studies have shown that chest stretching exercises increase the shoulder range of motion, lessen pain, and enhance function in SIS patients (Park et al., 2020). Such exercises can also improve thoracic kyphosis and reduce scapular protection (Yoo, 2013). Manual therapy focusing on chest flexibility has been found to improve shoulder function and decrease discomfort and kyphosis in SIS patients (Andrews et al., 2018). However, based on our current understanding, there is still a lack of sufficient research on the effectiveness of scapular stability training (SST) combined with thoracic flexibility training (TFT) for improving shoulder function and reducing SD in table tennis players. Therefore, this study aimed to compare the effects of SST alone versus combined SST and TFT on shoulder function in elite table tennis players with SD, and assess the impact of these training protocols on the scapular position, proprioception, and overall function.

## Methods

### Participants

Initially, 35 athletes were enrolled in this investigation. However, five individuals did not meet the inclusion criteria as two of them had undergone shoulder surgery and three others suffered from back pain. Ultimately 30 participants were retained. Ten individuals (5 males and 5 females) without scapular dysfunction composed the control group. The remaining twenty table tennis players were split into two groups using an online randomization tool (https://www.randomizer.org/): shoulder and chest (7 males, 3 females, and 5 males, 5 females, respectively). A pilot study recommended a group size of ten to identify a significant effect size (Park et al., 2020). Table 1 indicates that all groups had comparable demographics, and every participant completed the training intervention.

**Table 1:** Demographic data of participants in the three groups.

Group	Age(years)	Body height(cm)	Body mass(kg)	Training experience (years)	Training frequency(times/week)	Training duration(h/time)
Control group (n = 10)	20.55 ± 1.64	172.00 ± 8.91	63.55 ± 11.02	12.09 ± 2.96	4.09 ± 0.70	2.32 ± 0.56
Shoulder group (n = 10)	20.33 ± 1.73	174.67 ± 8.92	64.28 ± 10.18	12.33 ± 2.20	4.67 ± 1.12	2.06 ± 0.39
Chest group(n = 10)	20.70 ± 2.83	168.40 ± 7.62	60.00 ± 8.89	13.70 ± 3.14	4.60 ± 0.70	2.05 ± 0.28

The inclusion criteria were as follows: (1) athletes of national level 2 or higher, with at least 10 years of training experience, who had achieved third place or higher in regional competitions, participated in training at least three times per week, and employed a right-handed forehand topspin for fast-attack and loop combination plays; (2) table tennis players with positive results in the scapular assistance test (SAT), the scapular retraction test (SRT), and scapular dyskinesis tests (SDTs) (Pires and Camargo, 2018) with no symptoms; (3) all participants had a clear understanding of the purpose of the experiment and potential risks, volunteered to participate, and signed informed consent forms. The exclusion criteria for participants were as follows: a history of shoulder dislocation, previous surgery on the shoulder, back or neck in the past six months, symptoms of neck or back dysfunction, scapular fractures, scapular dysfunction caused by central or peripheral nerve injury, and other injuries or illnesses preventing from participation in this study.

### Measures

(1) MicroFET3 Muscle Strength and Spinal Range of Motion Measurement Device

Using the microFET3 device, participants stood with their hips at 90° flexion and their arms crossed. The device first measured the left spinal rotation ROM at T1 and then, while maintaining the position, measured the ROM at T12 after participants returned to the initial position. Thoracic spine ROM was calculated as ROM_T12_ − ROM_T1_.

(2) Upper Quarter Y-Balance Test (UQ-YBT) (Pires and Camargo, 2018)

The UQ-YBT evaluates upper limb motion and stability through the closed kinetic chain test. Participants began in a push-up position, extending one arm as far as possible in the forward, posterior-medial, and posterior-lateral directions and returning to start in each direction. This process was repeated three times per direction, and the average distance was recorded. The score was the sum of these distances divided by three times the length of the supporting limb. The NRS-2002 has excellent interrater reliability and a range of 0.80 to 0.99 for test-retest reliability (Gorman et al., 2012).

(3) Multi-Joint System (MJS) for Shoulder Joint Proprioception (Gorji et al., 2022)

The average track error (ATE) metric indicates the percentage deviation between the actual and set distances. Participants completed two practice trials, then performed three identical movements per test, with 30-s rest intervals between movements and 5-min rest intervals between sets.

(4) BTE Primus RS

The isokinetic maximum strength test set at 60°/s measured players' maximum strength during a forehand loop. In the force-velocity test, resistance was set at 25% of the maximum strength to mimic the forehand loop motion, measuring angular velocity and power.

(5) Lateral Scapular Slide Test (LSST)

The distance from the inferior angle of the scapular to the spinous process at the same level on both sides was measured three times using a ruler. Position 1: arms relaxed at the sides. Position 2: hands on the hips, humerus angled at 45° in the coronal plane. Position 3: arms abducted to 90°. A difference of 1.5 cm between the two sides indicated a positive test (Odom et al., 2001). Table tennis players may show greater LSST asymmetry due to frequent arm extension at play. Despite reliability issues (Odom et al., 2001), the clinical assessment method of the LSST is a useful tool for monitoring and measuring scapular asymmetry in clinical settings due to its simplicity.

### Design and Procedures

To mitigate the impact of fatigue on participants’ evaluation during the testing procedure, all assessments were conducted over two days. First, demographic and training history data were gathered. On day one, assessments included the lateral scapular slide test (LSST), the thoracic spine rotation range test, and the Y balance test (YBT). On day two, there was a 30-min rest interval between the multi-joint stability (MJS) test and the BTE Primus RSTM test. Participants underwent two practice trials before the measurements for the YBT, MJS, BTE Primus RSTM, and thoracic spine rotation tests. Testing order was randomized. Before the intervention, all three groups underwent all the tests. Since the control group did not receive any intervention, only the shoulder and chest groups underwent all tests again after the intervention, consistent with the pre-intervention tests.

The same staff conducted all the interventions. The shoulder group received only SST, while the chest group received SST for the playing side, TFT for both sides, and posterior to anterior thoracic mobilization. These interventions lasted six weeks, with three sessions a week, each lasting 30–40 min. The resistance of the elastic band increased every two weeks, starting with the yellow band (resistance: 3 lbs), moving to the green band (resistance: 4.6 lbs), and ultimately using the blue band (resistance: 5.8 lbs). Similarly, the volume of the thoracic training increased every two weeks, from 8 to 10 repetitions per set, for a total of 12 repetitions.

All interventions were supervised by the same group of physical therapists.

(1) Elastic Band Resistance TYW Exercise

**Figure 1:**
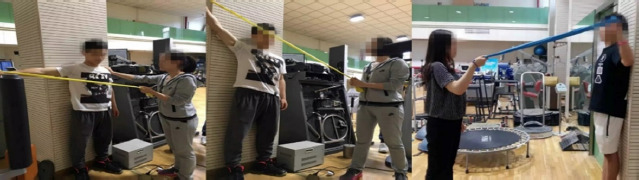
Resistance training with the elastic band in a TYW exercise.

T Position: The racket arm was held at 90°, and the elastic band was held to resist. Y Position: upper limb abducted at 145° to resist the elastic band. W Position: racket arm abducted at 90°, with the elbow flexed at 60° to resist the elastic band (Figure 1).

(2) External/Internal Rotation Resistance

**Figure 2:**
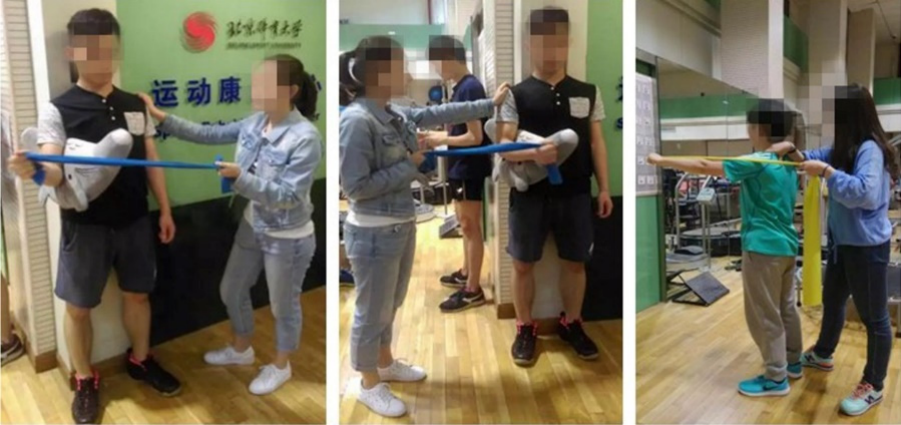
Resistance of the external rotation/internal rotation elastic band in the standing position.

The elbows were flexed at 90°C, resisting the elastic band for external and internal rotation of the shoulder joint and maintaining the position for 15 s before returning to the starting position (Figure 2).

(3) Serratus Anterior Punch with an Elastic Band

Elbows were extended and shoulders flexed forward at 90°, with the scapular protracted, maintaining the maximum protracted end position for 15 s, then returning to the starting position by stretching the band forward.

(4) Seated Trace the Circle (Balke et al., 2011)

**Figure 3:**
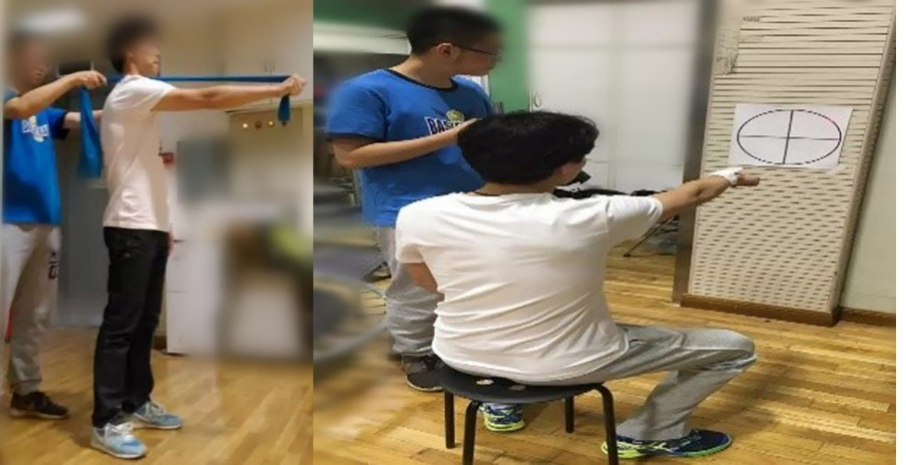
Serratus anterior muscle punch with an elastic band and drawing and tracking from the sitting position.

Participants were seated one meter from the wall, extended forward at shoulder level with their palms down and fists closed, and a laser pointer was used on one hand to trace a circle on a target board, which was adjusted to align with the scapulo-humeral joint (Figure 3). Two sets were performed with 30-s rest intervals.

(5) TFT Intervention

**Figure 4:**
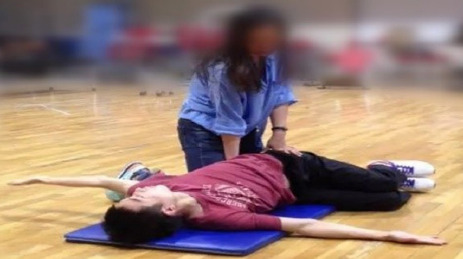
Rotation of the thoracic spine in the lateral position.

Participants lay on one side with their hips and knees flexed at 90°, their shoulders flexed at 90°, and their arms extended. Then, the arm rotated across the body, involving thoracic spine movement (Figure 4).

### Statistical Analysis

SPSS 22.0 was used for data analysis, and the mean ± standard deviation was used as the output. The Shapiro-Wilk test was applied to check for a normal distribution. Within-group paired *t*-tests were conducted for normally distributed data, while the Wilcoxon signed-rank test was applied for non-normally distributed data. Independent sample *t*-tests were carried out for between-group comparisons of normally distributed data, and the Mann-Whitney U test was used for non-normally distributed data. For three-group comparisons, one-way ANOVA was conducted for normally distributed data, and the Kruskal-Wallis test was used for non-normally distributed data. Statistical significance was set at *p* < 0.05.

## Results

From the data in Table 2, for the shoulder group, it was evident that ATE (%) on the playing side was significantly lower after the intervention compared to baseline (P50 pre-training and post-training values were 43.00, and 33.50, respectively; *p* = 0.005). The angular velocity (°/s) increased after the intervention compared to baseline, and the difference was statistically significant (P50 pre-training and post-training values were 440.15 and 455.70, respectively; *p* = 0.005). The LSSTs at position one (cm) (P50 pre-training = 0.67, P50 post-training = 0.29), position two (P50 pre-training = 0.87, P50 post-training = 0.52), and position three (P50 pre-training = 0.83, P50 post-training = 0.47) were all significantly smaller after the intervention when compared to baseline (*p* values were 0.005, 0.008, and 0.009, respectively).

**Table 2:** Results from different tests.

Variable	Shoulder group		Rank-sum Test		Chest group		Rank-sum Test	
	Pre-Median	Post-Median	Z	*p*	Pre-Median	Post-Median	Z	*p*
Playing-side Average Track Error (%)	43.00	33.50^△^	−2.807	0.005	42.50	32.00^△^	−2.803	0.005
Angle Velocity (°/s)	440.15	455.70^△^	−2.805	0.005	440.50	479.20^△^	−2.803	0.005
LSST Position1 (cm)	0.67	0.29	−2.805	0.005	0.35	0.37	−1.785	0.074
LSST Position2 (cm)	0.87	0.52^△^	−2.670	0.008	0.92	0.38^△^	−2.803	0.005
LSST Position3 (cm)	0.83	0.47^△^	−2.601	0.009	0.80	0.60^△^	−2.666	0.008
Nonplaying side YBT (%)	83.21	84.61	−1.362	0.173	78.55	90.08^△^	−2.803	0.005
Playing-side YBT (%)	79.53	82.03	−0.153	0.878	79.61	91.51^△^	−2.803	0.005
Thoracic rotation ROM_left_ (°)	26.33	24.83	−0.153	0.878	26.50	26.50	−0.612	0.541
Thoracic rotation ROM_right_ (°)	25.17	27.17	−0.210	0.833	22.92	28.17^△^	−2.701	0.007
CON_max_ (N)	163.00	164.50	−0.459	0.646	167.50	187.50	−0.866	0.386
ECC_max_ (N)	293.50	268.50	−0.255	0.799	276.00	263.00	−0.459	0.646
CON_ave_ (N)	140.90	159.35	−1.376	0.169	143.45	150.15	−0.968	0.333
ECC_ave_ (N)	241.40	210.95	−0.051	0.959	217.3	228.75	−0.663	0.508
ECC/CON_max_	1.52	1.46	−0.561	0.575	1.57	1.60	−0.459	0.646
ECC/CON_ave_	1.48	1.59	−0.357	0.721	1.52	1.45	−0.918	0.359

△ indicates significant differences between the results before and after the intervention (p < 0.05)

Regarding the chest group, both ATE and angular velocity on the playing side were significantly different before and after the intervention (*p* = 0.005). The LSSTs at positions two and three were both significantly smaller post intervention compared to baseline (*p* values were 0.005 and 0.008, respectively). Following the intervention, the non-playing side and playing-side YBT (%)increased compared to baseline, and these differences were significant (*p* = 0.005). Additionally, the average value of the ROM for thoracic rotation to the right increased post intervention (*p* = 0.007).

The chest group showed significant differences in angular velocity (difference of −29.00, 95% CI −56.30–−7.00, *p* = 0.003), playing-side YBT distance (difference of −9.63, 95% CI −15.11–−4.16, *p* = 0.002), and the average value of right thoracic rotation ROM (difference of −5.12, 95% CI −7.97–−2.27, *p* = 0.001) compared to those of the shoulder group. The other variables did not differ significantly (Table 3).

**Table 3:** Comparison of the differences between the pre- and post-intervention results.

Variable	Group	Mean ± SD/Median(P25,P75)	D(95%CI)/MD (95%CI)	T/Rank-sum Test	
				*t*/*Z*	*p*
Playing-side Average Track Error (%)	Shoulder group	−13.00 (−21.75, −1.75)	0.01(−11.00,8.00)	−0.040^b^	0.970
	Chest group	−10.50 (−17.50, −5.50)			
Angle Velocity (°/s)	Shoulder group	9.20 (4.93,2.95)	−29.00(−56.30,−7.00)	−2.950^b^	0.003
	Chest group	44.35 (24.53,86.75)*			
LSST Position 1 (cm)	Shoulder group	−0.17 (−0.28, −0.03)	0.02(−0.21,0.48)	−0.380^b^	0.705
	Chest group	−0.12 (−0.73, −0.05)			
LSST Position 2 (cm)	Shoulder group	−0.23 (−0.40, −0.17)	0.03(−0.17,0.49)	−0.300^b^	0.762
	Chest group	−0.27 (−0.80, −0.06)			
LSST Position 3 (cm)	Shoulder group	−0.29 (−0.62, −0.22)	−0.20(−0.40,0.07)	−1.740^b^	0.082
	Chest group	−0.09 (−0.32, −0.04)			
Non-playing side YBT (%)	Shoulder group	7.70 (−1.61,9.81)	−3.58(−13.38,3.15)	−1.060^b^	0.290
	Chest group	8.94 (4.43,14.19)			
Playing-side YBT (%)	Shoulder group	1.23 ± 5.92	−9.63(−15.11,−4.16)	−3.700^a^	0.002
	Chest group	10.87 ± 5.73*			
Thoracic rotation ROM_left_ (°)	Shoulder group	0.10 ± 3.10	−1.47(−5.95,3.02)	−0.690^a^	0.501
	Chest group	1.57 ± 6.00			
Thoracic rotation ROM_right_ (°)	Shoulder group	−0.40 ± 2.8	−5.12(−7.97,−2.27)	−3.770^a^	0.001
	Chest group	4.72 ± 3.25*			
	Chest group	−1.95 ± 1.68			
CON_max_ (N)	Shoulder group	4.70 ± 62.67	−9.70(−65.48,46.08)	−0.37^a^	0.719
	Chest group	14.40 ± 55.86			
ECC_max_ (N)	Shoulder group	4.70 ± 62.67			
	Chest group	14.40 ± 55.86			
CON_ave_ (N)	Shoulder group	16.40 ± 31.81	3.22(−32.08,38.52)	0.19^a^	0.85
	Chest group	13.18 ± 42.57			
ECC_ave_ (N)	Shoulder group	3.76 ± 67.00	−5.05(−63.23,53.13)	−0.18^a^	0.857
	Chest group	8.81 ± 56.38			
ECC/CON_max_	Shoulder group	−0.04 (−0.89,0.15)	−0.01(−0.76,0.22)	0.01^b^	0.99
	Chest group	−0.08 (−0.17,0.21)			
ECC/CON_ave_	Shoulder group	−0.04 (−0.32,0.30)	0.09(−0.31,0.49)	−0.42^b^	0.677
	Chest group	−0.13 (−0.38, 0.20)			

CI: confidence interval; *: significant difference compared to the shoulder group (p < 0.05). Difference = Results_Post-test_ − Results_Pre-test_. MD: median difference

Prior to the intervention, the shoulder group showed significant differences compared to the control group in ATE, angular velocity, and position three, with *p*-values of 0.039, 0.025, and 0.015, respectively. Similarly, the chest group exhibited significant differences compared to the control group in angular velocity and position three, with *p*-values of 0.043, and 0.030 (Table 4).

**Table 4:** Results from different tests in three groups (pre-test).

Variable	Group	Mean ± SD/Median	CI/(P25,P75)	F/H test	
				F/H	*p*
Playing-side Average Track Error (%)	Control group	32.00^1)^	29.00,39.25	7.849^d^	0.020^α^0.039^β^0.054^γ^
	Shoulder group	43.00^1)#^	36.75,50.25		
	Chest group	42.50^1)#^	33.00,51.50		
Angle Velocity (°/s)	Control group	457.00^1)^	446.50,493.00	6.125^d^	0.047^α^0.025^β^0.043^γ^
	Shoulder group	440.15^1)#^	429.42,448.75		
	Chest group	440.50^1)#^	350.50,460.75		
LSST Position 1 (cm)	Control group	0.25^1)^	0.09,0.56	4.736^d^	0.094^α^
	Shoulder group	0.67^1)^	0.35,1.34		
	Chest group	0.35^1)^	0.16,1.40		
LSST Position 2 (cm)	Control group	0.47 ± 0.25	0.32,0.62	3.281^c^	0.053^α^
	Shoulder group	0.84 ± 0.48	0.55,1.13		
	Chest group	1.06 ± 0.72	0.66,1.58		
LSST Position 3 (cm)	Control group	0.35 ± 0.25	0.21,0.52	4.048^c^	0.029^α^0.015^β^0.030^γ^
	Shoulder group	0.91 ± 0.62^#^	0.57,1.34		
	Chest group	0.84 ± 0.49^#^	0.56,1.13		
Non-playing side YBT (%)	Control group	80.23 ± 7.3	75.84,84.77	0.043^c^	0.958^α^
	Shoulder group	79.97 ± 9.91	73.49,85.56		
	Chest group	79.07 ± 10.49	72.57,85.45		
Playing-side YBT (%)	Control group	86.41 ± 9.00	81.13,92.09	2.316^c^	0.118^α^
	Shoulder group	78.88 ± 10.05	72.82,84.86		
	Chest group	78.31 ± 9.10	72.55,84.27		
Thoracic rotation ROM_left_ (°)	Control group	23.73 ± 4.49	21.00,26.43	1.489^c^	0.243^α^
	Shoulder group	26.43 ± 2.90	24.75,28.36		
	Chest group	25.77 ± 3.36	23.58,27.81		
Thoracic rotation ROM_right_ (°)	Control group	23.07 ± 4.26	20.43,25.75	0.860^c^	0.435^α^
	Shoulder group	25.40 ± 3.37	23.33,27.53		
	Chest group	24.18 ± 4.25	21.47,27.00		
CON_max_ (N)	Control group	176.50 ± 26.80	161.35,194.99	0.029^c^	0.972^α^
	Shoulder group	173.10 ± 36.92	152.10,196.75		
	Chest group	176.20 ± 40.48	151.17,201.31		
ECC_max_ (N)	Control group	226.10 ± 50.14	197.01,259.28	2.990^c^	0.067^α^
	Shoulder group	286.50 ± 70.47	245.28,330.70		
	Chest group	270.70 ± 48.62	241.34,302.15		
CON_ave_ (N)	Control group	153.05 ± 19.60	141.85,165.35	0.089^c^	0.915^α^
	Shoulder group	147.57 ± 39.80	121.81,171.47		
	Chest group	147.30 ± 39.91	123.60,173.65		
ECC_ave_ (N)	Control group	217.55^1)^	195.48,328.88	0.359^d^	0.836^α^
	Shoulder group	241.40^1)^	167.88,269.82		
	Chest group	217.30^1)^	190.83,271.88		
ECC/CON_max_	Control group	1.30±0.29	1.12,1.47	3.315^c^	0.052^α^
	Shoulder group	1.69±0.48	1.43,2.00		
	Chest group	1.56±0.22	1.43,1.70		
ECC/CON_ave_	Control group	1.61^1)^	11.34,1.87	0.304^d^	0.859^α^
	Shoulder group	1.48^1)^	1.34,1.78		
	Chest group	1.52^1)^	1.32,1.83		

1): median; ^c^: F value; ^d^: H value; ^α^: the overall p-value for the comparison among the three groups; ^β^: p-value for the comparison between the shoulder group and the control group; ^γ^: p-value for the comparison between the chest group and the control group

## Discussion

This study indicates that SST combined with TFT contributes to improvements in proprioception, the scapular position, stability, and performance in table tennis players with SD. However, combining SST with TFT may be more advantageous for enhancing thoracic flexibility and the flexibility of the playing-side limbs in table tennis players. This combination appears to have a more pronounced impact on the increase in hitting speed and improvement in motor skills compared to isolated SST.

### Proprioception

This study assessed ATE in players' playing-side scapulas and revealed improved proprioception in both the shoulder and chest groups, with a significant reduction in ATE post intervention (*p* = 0.05), indicating training benefits for proprioceptive abilities. This finding is in contrast to earlier studies that found no gains in proprioception with comparable training (Shang et al., 2022). Our results suggest that strength and proprioception pathways may differ, highlighting the role of muscle-neural coordination and joint proprioceptive changes in proprioceptive abilities (Myers and Lephart, 2000).

We also found improved shoulder proprioceptive abilities despite no increase in maximum concentric (CON) and eccentric (ECC) strength. This is consistent with research suggesting a relationship between proprioception and strength, especially post injury (Machner et al., 2003). Macefield et al. (2013) indicated that targeted and controlled training reduced ATE of the playing side scapula, most likely as a result of improved proprioception and muscle nerve sensitivity.

This study revealed increased angular velocity in forehand topspin strokes, indicating improved movement coordination. This improvement in proprioception supports the idea that focused scapular controlled training boosts proprioceptive abilities (Mottram, 1997). However, additional studies are needed to determine the causes of this proprioceptive improvement.

### Positioning, Flexibility, and Performance

Table tennis requires players to make rapid decisions and react quickly within 0.2 to 0.4 s (Bankosz and Barczyk-Pawelec, 2020), making increased ball speed a key factor in enhancing competitive performance. This study's angular velocity assessment showed that post-training, the chest group had a greater mean angular velocity during hits than did the shoulder group, suggesting improved movement performance. Training focused on slow, controlled movements, engaging slow-to-conduct, fatigue-resistant small muscle fibers (Walker et al., 2017) may account for the increased hitting speed without a corresponding strength increase.

According to our study, improving thoracic spine flexibility could enhance grip strength and speed. This could account for performance gains associated with TFT. Shoulder flexion, adduction, internal rotation, and elbow pronation are the dominant actions in forehand topspin strokes (Li et al., 2021). Research shows that table tennis players often lack shoulder internal rotation during topspin strokes, potentially restricting their range of motion (Zhang et al., 2023). Biomechanical analysis revealed that table tennis players have increased internal rotation torque and angular velocity in the shoulder joint, which are crucial for performance (Li et al., 2021). Our intervention led to enhanced thoracic spine rotation, aiding glenohumeral joint internal rotation and potentially improving hitting power and speed.

Training also addressed postural issues such as thoracic kyphosis and anterior cervical bending common in players, which can lead to SD (Longo et al., 2020). Lower trapezius activation and thoracic kyphosis correction are the goals of exercise, such as joint mobilization (Park et al., 2020), YTW exercise, and pectoralis minor stretching (Borstad and Ludewig, 2006). Pectoralis minor flexibility is enhanced by training between 90° and 150° of arm abduction, which helps in scapular external rotation and posterior tilting (Longo et al., 2020). The increased YBT in the chest group can be explained by the presence of the serratus anterior, which also helps stabilize the scapular's inner edge, resist chest movement, and facilitate the action of scapular external rotators (Kibler et al., 2012). By realigning the thoracic spine, we enhanced posture and the overall athletic performance.

In contrast to athletes with normal function, those with SD had greater mean values at LSST position three, indicating a greater difference in scapular external rotation according to baseline comparisons. Following training, there were notable changes in both groups' scapular postures at two and three years, indicating improved balance and perhaps affecting the activation of the scapular muscles and surrounding soft tissue (Walker et al., 2017). The enhanced angular velocity and sports performance may be explained by these modifications. SD occurs when the typical position and movement of the scapula are significantly disrupted, leading to atypical and inefficient movements of the arm and shoulder (Jildeh et al., 2021). The need for external rotation support during forehead topspin motion may also contribute to improved performance. Overall, this study highlights the connection between shoulder joint function and scapular position correction, which raises the question of how scapular alterations affect sports skills and biomechanics. Although scapular mobility is strongly impacted by exercise, the best way to address scapular dysfunction has yet to be identified (Nodehi Moghadam et al., 2020).

### Stability

The YBT on the dominant side in the chest group increased significantly, which aligns with our previous analysis highlighting increased flexibility in the chest group. Additionally, the non-dominant side of the chest group showed significant improvement after the intervention compared to baseline, emphasizing enhanced stability in the playing side. Although there was no significant difference, the shoulder group showed an increase in the YBT during support on both the playing and non-playing sides.

The efficient and rapid execution of movements by athletes depends on the activation of stable muscles (Sekulic et al., 2013). Physiologically and biomechanically (Kibler et al., 2012), optimal scapular function plays a crucial role in shoulder movements, providing a stable foundation for the coupling and coordinated motion between the shoulder and the arm. Disruptions in this kinetic chain, often due to insufficient strength and activation, can lead to movement dysregulation and functional impairment (Pires and Camargo, 2018).

Shoulder instability can result from abnormal scapular motion, and subacromial impingement risk is associated with scapular angle changes during arm lifting (Kibler and Sciascia, 2016). These abnormalities can affect scapular stability and cause the anterior tilt, downward rotation, and poor activation of the serratus anterior and lower trapezius muscles (Lopes et al., 2015). Thus, it is essential to strengthen muscles such as the serratus anterior and lower trapezius to stabilize scapular mobility (Kibler et al., 2012). This study focused on stability training and improved the scapular position and function, although no significant changes were observed in the shoulder group, suggesting that enhancing scapular function may require multidimensional and directional training. Even though changes were not significant in either supported position (dominant and non-dominant) in the shoulder group, the training effects found in the chest group seem to support this evidence.

The chest group's left and right thoracic spine rotations improved considerably after training, demonstrating that TFT and joint mobilization improve rotation. Table tennis players are generally kyphotic. Joint mobilization is beneficial in correcting thoracic sagittal alignment, enhancing thoracic extension, and neutralizing the athlete's spine (Maitland et al., 2005; Powers et al., 2003). Biomechanically, maintaining a neutral spine position may improve rotational motion by increasing spine mobility in the other two directions. Thoracic spine active rotations (TFT) to the left and right may improve rotational mobility even more. According to our study, flexibility training in the chest group might increase the upward rotation of the scapula on the playing side. Those factors contributed to the improvement in the YBT. However, previous research has presented conflicting results regarding the effects of therapeutic exercise on scapular positioning and movement (van Tulder et al., 2003). This training may have also optimized the position of the scapula on the non-dominant side through overall thoracic spine adjustment in the sagittal plane, even though the non-dominant side muscles did not receive stability training. Such positioning could enhance activation of the surrounding muscles, providing a more stable base. Additionally, research suggests that strengthening the rotator cuff with the scapula in a neutral, retracted position can increase strength by 13% to 24% (Kibler et al., 2012); however, further study is needed to confirm this theory.

Both groups showed an increase in YBT scores, indicating that our training program may strengthen scapular stability in the dominant side. Overall, our training program contributed to improved scapular positioning and enhanced neuromuscular control of surrounding muscles, offering initial evidence of the effectiveness of stability training in boosting athletic performance.

### Limitations and Future Research

This study indicated that a comprehensive program addressing both scapular stability and thoracic mobility could significantly benefit table tennis athletes with shoulder issues. Practitioners might consider integrating such dual-focus interventions into training to enhance performance and reduce injury risks. However, the small sample size restricts the depth of the analysis and its applicability to varying age groups and proficiency levels. The absence of blinding for therapists and participants could introduce bias in evaluating the interventions' effects. Future research should examine the specific impacts of scapular and thoracic training on athletes with concurrent shoulder pain and scapular dysfunction, aiming to refine training protocols more effectively.

## Conclusions

The present study examined the impact of SST and combined training of SST and TFT. Specifically, the findings indicate that SST significantly improved proprioception and scapular stability in athletes with scapular dyskinesis. Moreover, combined training not only significantly enhanced proprioception and stability, but also notably increased angular velocity and thoracic rotation. This suggests that combined training has greater potential in enhancing the overall athletic performance of table tennis players.
